# 904. Racemic Epinephrine Nebulization in Pediatric Patients with Inhalation Injury

**DOI:** 10.1093/jbcr/irag033.408

**Published:** 2026-04-06

**Authors:** Vickie R Walker, Jong Lee

**Affiliations:** Shriners Children's - Texas, League City, TX; Division of Burn and Trauma Surgery, Department of Surgery, University of Texas Medical Branch, Galveston, TX

## Abstract

**Introduction:**

Successful treatment of pediatric inhalation injuries depends on preventing airway occlusion and avoiding intubation, as children have smaller airways that are more prone to edema and long-term complications such as subglottic stenosis. A single pharmacological agent that can decrease bronchospasm, reduce airway edema, and limit airway transudation would therefore be highly valuable. Nebulized racemic epinephrine, a mixture of both enantiomers of epinephrine, has been used for inhalation injury due to its combined bronchodilator and vasoconstrictive effects. These mechanisms may reduce airway obstruction, mucosal edema, and secretion volume, distinguishing it from other bronchodilators in this setting. Epinephrine exerts chronotropic effects through beta-1 adrenergic stimulation, heart rate (HR) is routinely monitored and was selected as the grouping variable to assess correlation with clinical outcomes. A decrease in HR may reflect improved airway stability and reduced work of breathing, whereas an increase may indicate instability. The aim of this study was to analyze clinical findings of patients receiving racemic epinephrine with standard of care (SOC) for inhalation injury, focusing on HR changes after treatment.

**Methods:**

A retrospective study was conducted of pediatric patients (n = 29; 0–17 years) with documented inhalation injuries who received nebulized racemic epinephrine along with SOC. Data collected included demographics, total body surface area (TBSA) burn, racemic epinephrine doses, HR response, length of stay, mortality, and the need for intubation. HR response was defined as an increase or decrease of >10% from baseline, with changes ≤10% considered unchanged. Descriptive statistics, ANOVA, and Fisher’s exact tests compared demographic, clinical, and outcome variables across HR groups.

**Results:**

HR decreased in 28% (n = 8), remained unchanged in 66% (n = 19), and increased in 7% (n = 2). No significant differences were found between groups for age (mean 6.0–8.1 years, *p*=.3), TBSA burn (mean 56.8–62.5%, *p*=.3), doses administered (mean 4.0–6.5, *p*=.7), length of stay (mean 54–92 days, *p*=.3), sex (*p*=.2), or mortality (*p*=1.0). A significant difference was observed in the rate of intubation (*p*=.03), with 13% of patients with a HR decrease versus 58% with unchanged HR requiring intubation.

**Conclusions:**

Nebulized racemic epinephrine appears effective. Most patients had no HR change; those with HR decrease were less likely to require intubation, suggesting improved airway stability. These findings align with its dual mechanism of action—beta-2 bronchodilation and alpha-1 vasoconstriction—Supporting its role as an adjunctive therapy. Prospective studies are needed to guide dosing and confirm efficacy.

**Applicability of Research to Practice:**

Racemic epinephrine may reduce airway edema, secretions, obstruction, and intubation in inhalation injury; studies needed to guide dosing.

**Funding for the study:**

N/A.

